# A case of pulmonary mucosa-associated lymphoid tissue lymphoma with plasmacytic differentiation and amyloid deposition: case report and literature review

**DOI:** 10.3389/fonc.2026.1718760

**Published:** 2026-03-05

**Authors:** Zhihui Wang, Xiaoxi Wang, Dingrong Zhong

**Affiliations:** China-Japan Friendship Hospital (Institute of Clinical Medical Sciences), Chinese Academy of Medical Sciences & Peking Union Medical College, Beijing, China

**Keywords:** histopathology, immunohistochemistry, lymphoma, MALT, pathology

## Abstract

Mucosa-associated lymphoid tissue (MALT) lymphoma is a relatively uncommon subtype of non-Hodgkin lymphoma which occurs anywhere but lymph nodes. We present a case of MALT lymphoma with plasmacytic differentiation and amyloid deposition. Although plasmacytic differentiation is shown in some cases, amyloid deposition is the rare one in the current cases we collected. Amyloidosis is often associated with some malignant diseases, but MALT is known as an indolent lymphoma. We want to report this case to raise pathologists’ cognizance on this disease.

## Introduction

Mucosa-associated lymphoid tissue (MALT) lymphoma is a relatively uncommon subtype of non-Hodgkin lymphoma, most frequently arising in the ocular adnexa, gastrointestinal tract, and salivary glands (1, 2). The precise pathogenesis remains unclear but is thought to be influenced by chronic infections (e.g., Helicobacter pylori), autoimmune disorders such as Sjögren’s syndrome and Hashimoto’s thyroiditis, and site-specific anatomical factors (3). Currently, there is no universally accepted staging system for MALT lymphoma, although accurate staging is critical for guiding therapy and predicting prognosis (4). Histological features, including plasmacytic differentiation, have been reported to correlate with clinical behavior. Here, we present a rare case of pulmonary MALT lymphoma with plasmacytic differentiation and amyloid deposition, accompanied by a review of relevant literature, aiming to expand current understanding of this disease.

## Case report

### Clinical presentation

A 51-year-old man with a history of bronchial asthma underwent routine imaging in 2021, which revealed incidental pulmonary lesions. No significant radiographic progression over 5 years. In 2025, follow-up image revealed a cystic lesion in the right superior lobe. The patient was asymptomatic, but imaging features raised concern for malignancy. No significant changes on physical examination. Clinical test results: WBC 6.88 × 10^9^/L, ProGRP 73.84 pg/ml. There was no prior history of autoimmune disease, immunosuppressive therapy, or organ transplantation. The patient recovered well after surgery and underwent regular follow-up CT scans.

### Past medical history

The patient had a three-year history of hypertension, controlled with irbesartan (0.5 mg daily), a >10-year history of untreated tachycardia, and a three-year history of asthma treated with budesonide and formoterol.

### Radiological findings

**Figure 1:**
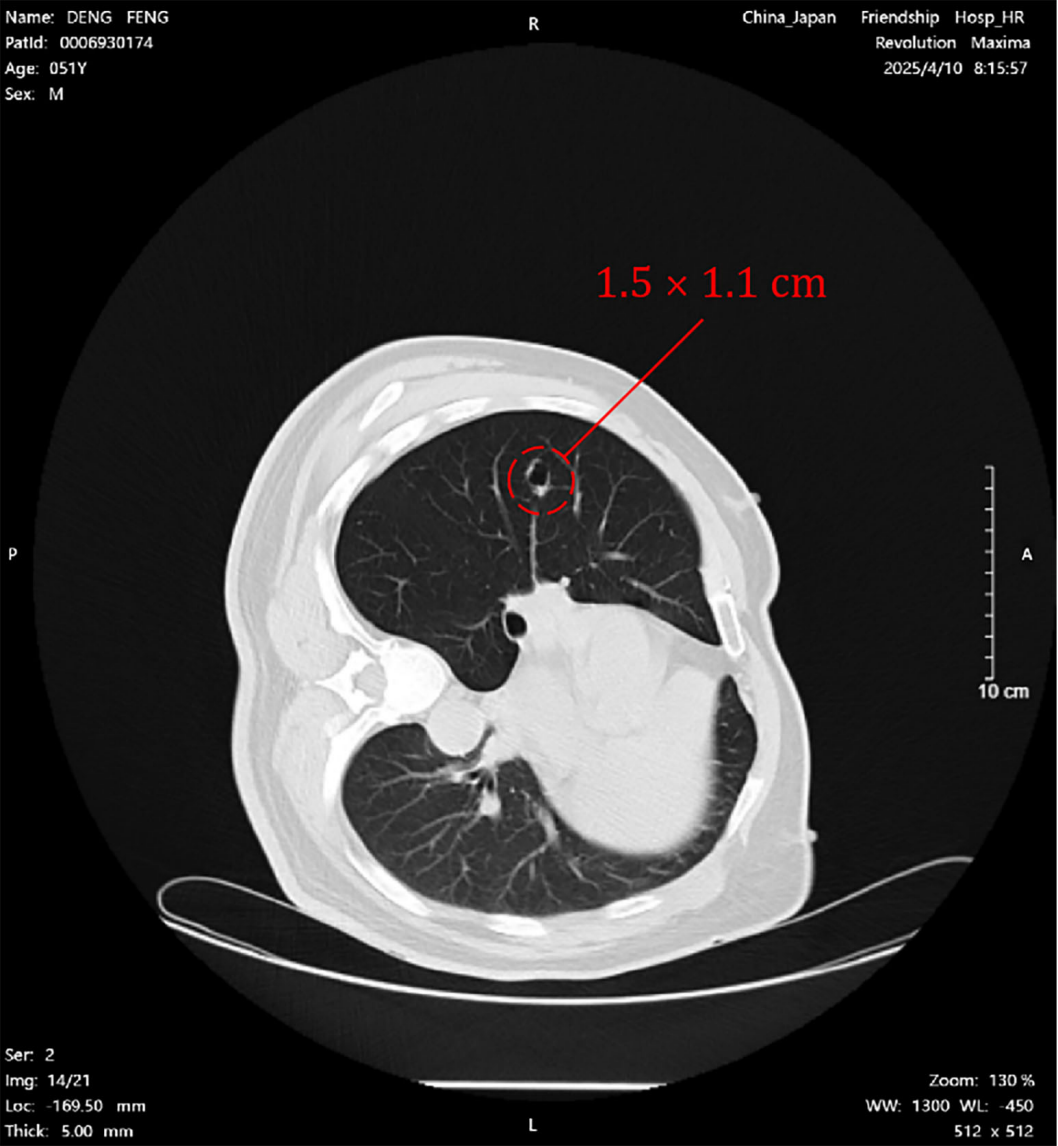
Chest PET-CT revealed a cystic cavity, measuring approximately 1.5 cm × 1.1 cm.

Positron emission tomography-computed tomography (PET-CT) performed in February 2025 at the China-Japan Friendship Hospital demonstrated a thin-walled cystic lesion in the right upper lobe containing a mural nodule measuring 1.5 × 1.1 cm, with mild fluorodeoxyglucose uptake (SUVmax 1.6) (**Figure 1**).

## Materials and methods

Surgically resected lung tissue was fixed in 4% neutral buffered formalin, paraffin-embedded, and sectioned for hematoxylin and eosin (H&E)(Beijing Yili Fine Chemicals Co,ltd) staining. Immunohistochemistry (IHC) was performed using the EnVision two-step method. Antibodies included CD138, Ki67 (Beijing Zhongshan Golgen Bridge Biological Technology Co., ltd), CD3, CD21, CD23, CK, BCL6, κ, λ (Fuzhou Maixin Biotech,ltd.), CD20, CD79α, CD5, CD10, BCL2, and Cyclin D1 (f. hoffmann-la roche, ltd). Special stains [Congo red (made by our lab), PTAH (Beijing Yili Fine Chemicals Co,ltd)] were also applied.

## Results

### Gross findings

**Figure 2:**
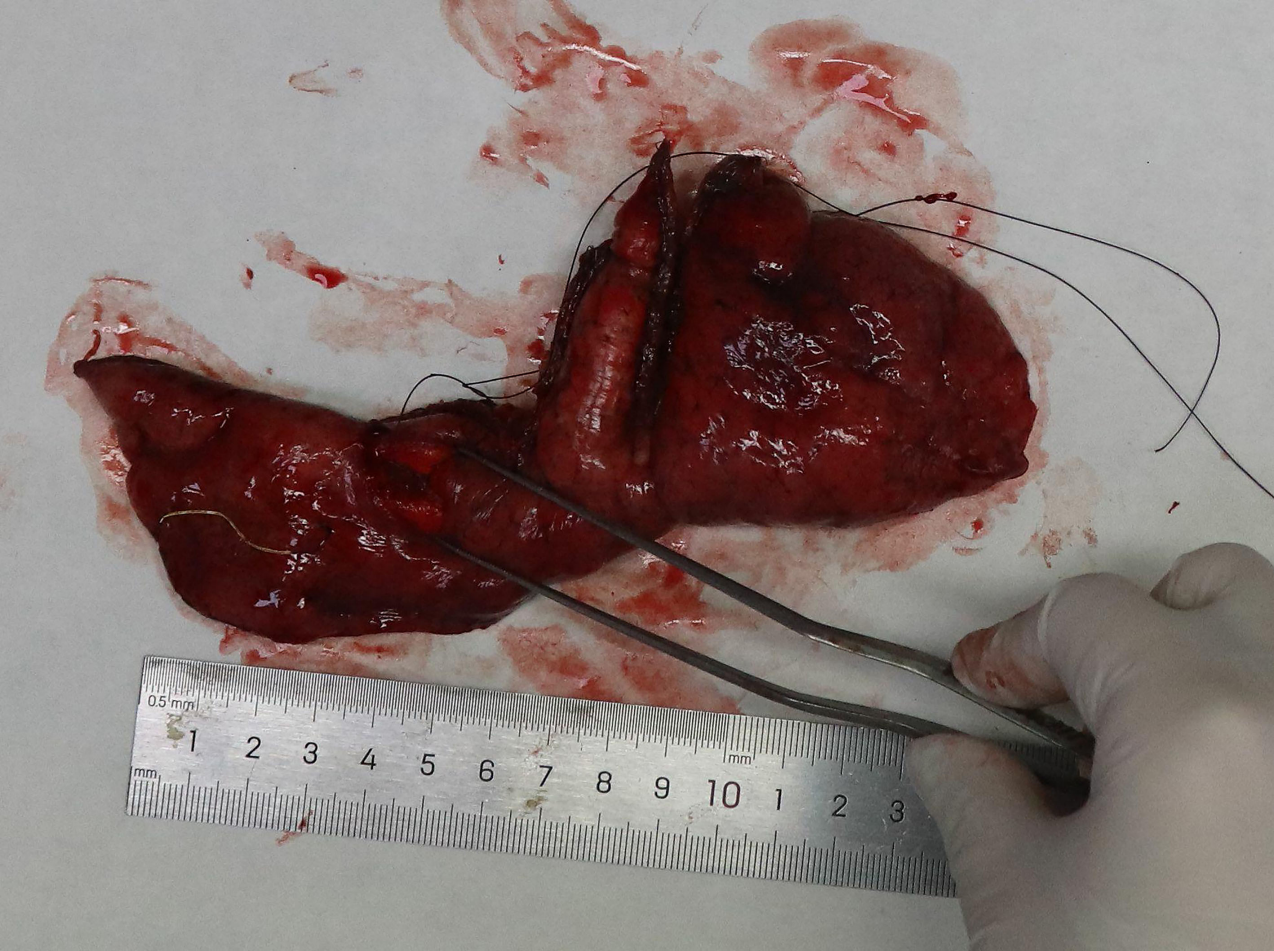
Segmentectomy specimen of the lung; a gray-white solid nodule is on the cut surface.

The lung segmentectomy specimen measured 16.0 × 7.0 × 2.0 cm. A solitary gray-white solid nodule, 1.5 × 1.0 × 0.8 cm in size, was located 1.4 cm from the staple line and 0.5 cm from the pleural margin (**Figure 2**). The lesion was well-demarcated without hemorrhage or necrosis, consistent with a neoplastic process.

### Microscopic findings

**Figure 3:**
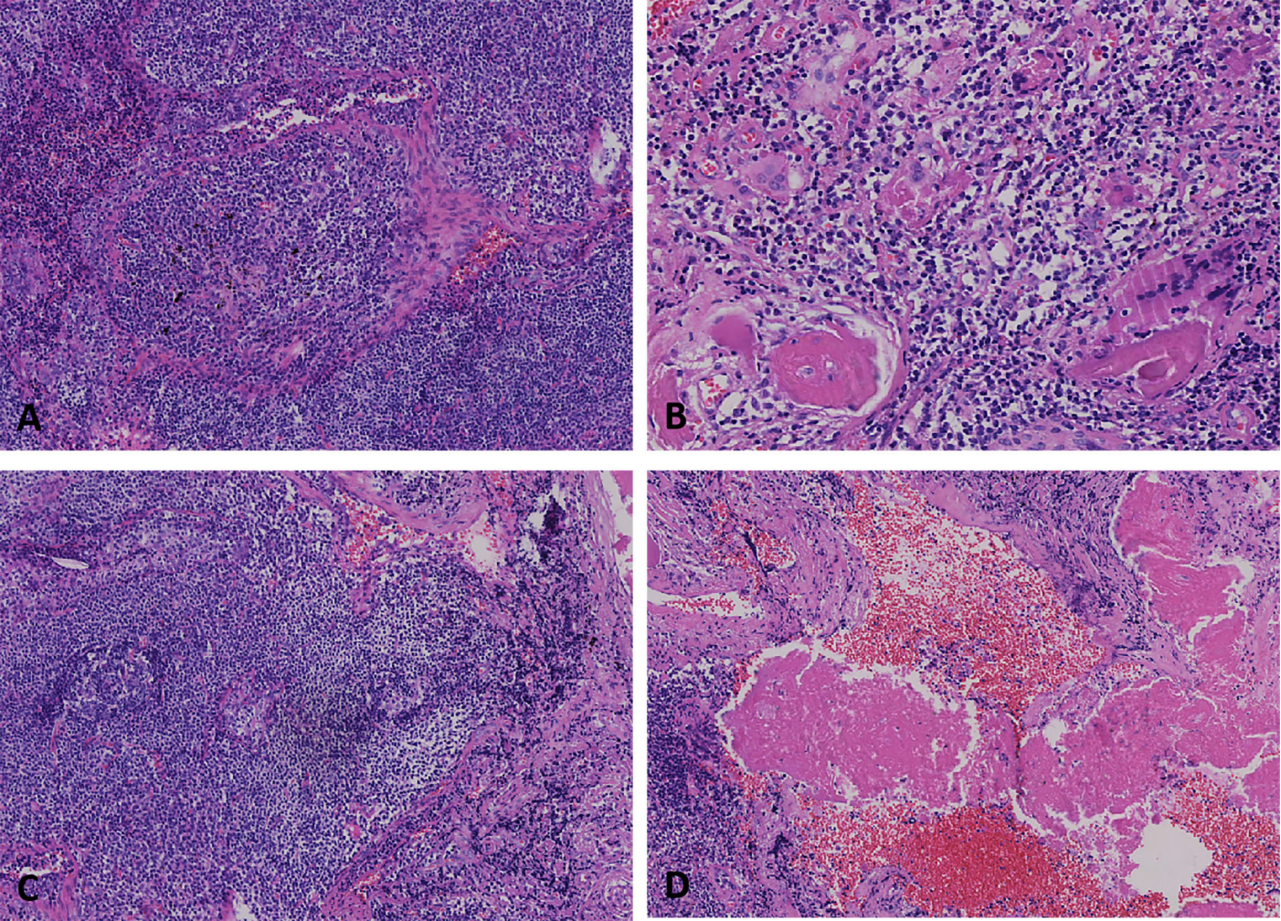
**(A)** Tumor cell infiltration forming lymphoepithelial lesions involving alveolar epithelial structures (H&E, ×20). **(B)** Foreign-body giant cell reaction with Dutcher bodies adjacent to tumor cells (H&E, ×20). **(C)** Plasmacytoid cell infiltration within follicular dendritic cell networks, consistent with reactive follicular hyperplasia (H&E, ×20). **(D)** Homogeneous eosinophilic amyloid deposits surrounding perivascular regions, associated with plasma cell infiltration (H&E, ×20).

Histology revealed diffuse infiltration of small-to-medium lymphoid cells with nodular and sheet-like architecture. Tumor cells displayed abundant cytoplasm, mildly irregular hyperchromatic nuclei, and conspicuous nucleoli. Characteristic lymphoepithelial lesions were observed, with neoplastic infiltration of alveolar epithelial structures. The lesion periphery demonstrated marginal zone-type monocytoid B cells and foreign-body giant cell reactions. Reactive follicular hyperplasia with plasmacytoid cell infiltration was noted within follicular dendritic networks. Homogeneous eosinophilic amyloid deposits were identified, predominantly in perivascular regions, associated with plasma cell infiltrates (**Figure 3**).

### Immunophenotypic profile and special staining results

**Figure 4:**
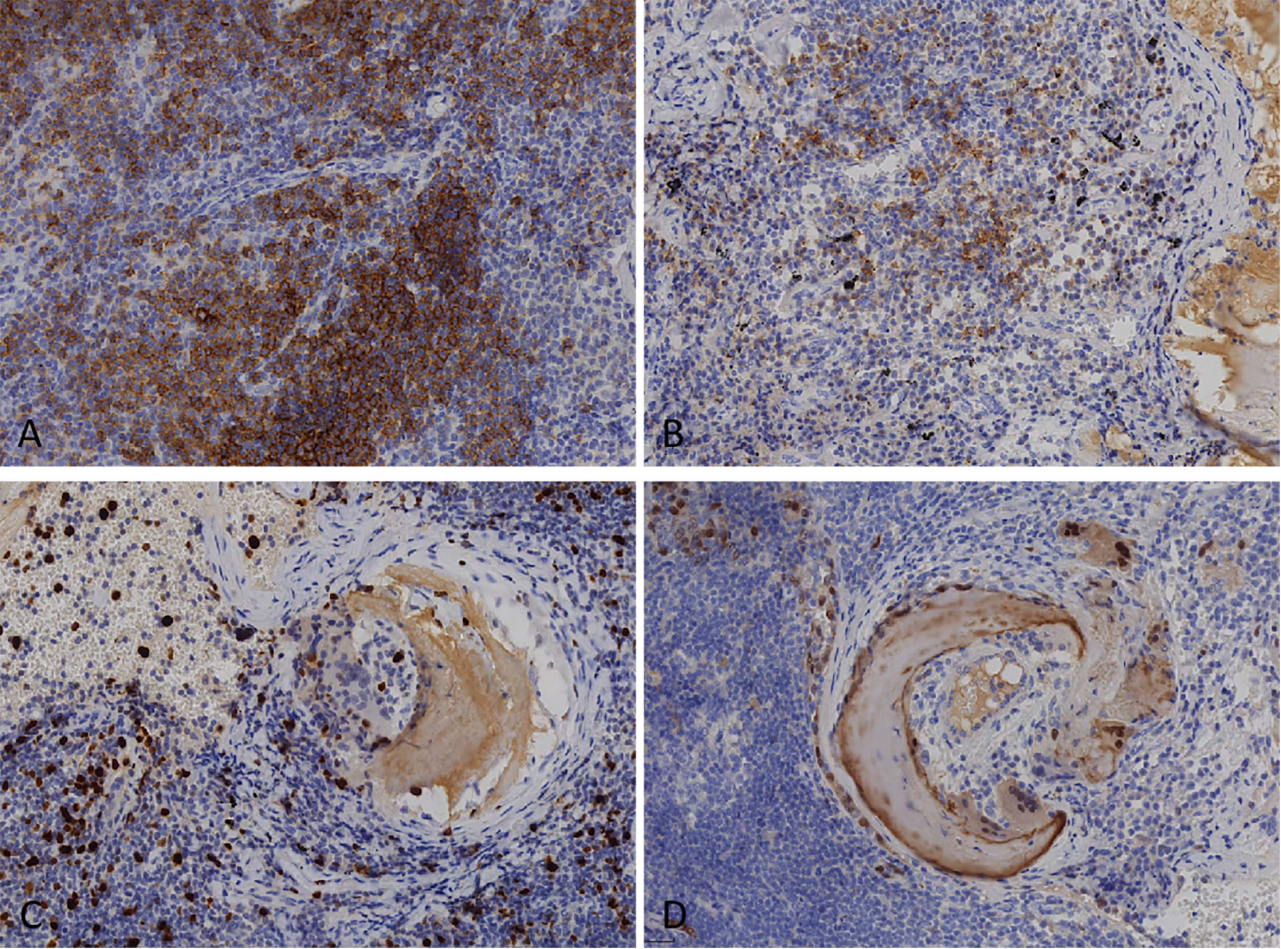
**(A)** CD21 immunostaining highlighting follicular dendritic cell meshworks (×20). **(B)** Strong membranous positivity of tumor cells for CD138 (×20). **(C)** Ki67 labeling index estimated at ~15%, indicating low proliferative activity (×20). **(D)** Cyclin D1 negative in neoplastic cells, excluding mantle cell lymphoma (×20).

Neoplastic cells expressed CD20, CD79α, BCL2, BCL6, and CD138, and were negative for CD3, CD5, and Cyclin D1. The Ki67 index was 10–20%, consistent with low proliferative activity. CD21 and CD23 highlighted reactive follicular dendritic cell networks(**Figure 4**). Congo red staining showed apple-green birefringence under polarized light microscopy. These findings supported a diagnosis of MALT lymphoma with plasmacytic differentiation and amyloid deposition.

### Molecular finding

Molecular detection shows no evidence of mutation of KRAS, NRAS and arrangement of TCRG, TCRD and TCRB. Whereas, it shows arrangement of IgH, IgK, IgL.

## Discussion

### Clinical characteristics

First described in 1983 by Isaacson and Wright, MALT lymphoma accounts for ~7–8% of all lymphomas (5). Although it can occur in various extranodal sites, pulmonary MALT lymphoma remains rare. It typically follows an indolent course and is often detected incidentally on imaging (6). Accurate diagnosis relies on integrating radiologic, histologic, and immunophenotypic findings. Surgical resection is often the primary treatment option for patients, and according to guidelines, there is no significant prognostic difference between resection alone and resection combined with radiotherapy or chemotherapy (3).

### Pathological characteristics

MALT lymphoma with plasmacytic differentiation is histologically defined by ≥10% of tumor cells exhibiting plasma cell-like morphology (7), including eccentric nuclei, basophilic cytoplasm, and Dutcher or Russell bodies. According to the features of plasma-like cell, we think amyloid deposition is a rare phenotype related to plasmacytic differentiation.

### Differential diagnosis

Key differentials include Plasma cell-type Castleman disease (PCD), mantle cell lymphoma (MCL), pulmonary nodular lymphoid hyperplasia (PNLH) and Lymphoplasmacytic Lymphoma (LPL). PCD is characterized by hyperplastic follicles, interfollicular plasma cell sheets, systemic manifestations, and frequent HHV8 association (8). MCL shows typically aggressive, with Cyclin D1 and SOX11 positivity and the hallmark t(11;14)(q13;q32) translocation (9, 10). PNLH has benign lymphoid proliferation with well-formed follicles and absence of significant plasmacytic infiltration, which radiologically presents as diffuse reticulonodular infiltrates rather than discrete nodules (11). Most of LPL cases show the MYD88 L265P mutation, but this mutation is not diagnostic for LPL, as it can also be expressed in MALT lymphoma rarely. The integration of morphology, IHC, and clinical findings in this case excluded these entities, confirming the diagnosis.

## Conclusion

We report a rare case of pulmonary MALT lymphoma with plasmacytic differentiation and amyloid deposition. Recognition of such features is essential to avoid misdiagnosis with other plasma cell-rich or reactive lymphoproliferative disorders. This case underscores the diagnostic value of combined histopathological and immunophenotypic assessment and contributes to the limited literature on pulmonary MALT lymphoma with amyloid production.

## Data Availability

The original contributions presented in the study are included in the article/Supplementary Material. Further inquiries can be directed to the corresponding author.
